# Success of a urinary catheter insertion team in reducing urinary infections in intensive care unit

**DOI:** 10.1186/2197-425X-3-S1-A893

**Published:** 2015-10-01

**Authors:** DA Regagnin, LR Guastelli, DS Alves

**Affiliations:** Intensive Care Unit, Hospital Israelita Albert Einstein, São Paulo, Brazil; Step Down Unit, Hospital Israelita Albert Einstein, São Paulo, Brazil

## Introduction

About 8-21% of hospital infections in intensive care units (ICU) are urinary [1, 2] being 80% of them associated with the use of urinary catheters [3]. Several studies show that the early removal of urinary catheters reduces the rate of urinary tract infection. However, critically ill patients who require this device do not have the option to remove it. For this group, the best preventive measure seems to be educative activity for the nursing staff responsible for the insertion and manipulation of this device.

## Objectives

Create a team of professionals trained to the insertion of urinary catheters and organize actions aimed at reducing the rate of urinary tract infection associated with urinary catheters in the intensive care unit.

## Methods

Prospective study conducted for 12 months in intensive care unit. Started in July 2013, the intervention program involved the creation of a qualified team for the insertion of urinary catheter and the creation of audits to stimulate the removal of inappropriate urinary catheters and assess the process of inserting these devices. The obtained results were compared with the 12 months preceding the beginning of the interventions.

## Results

The comparison between the months of August 2012 to July 2013 and August 2013 to July 2014 shows that there was a fall of 60% (2.4-1.0 p = 0.040) in the rate of urinary tract infection associated with urinary catheter and a reduction of 13.7% (from 0.24 to 0.21 p = 0.001) in the utilization rate of urinary catheters. In the twelve months of intervention it was also noted a trend of reducing the number of urinary catheters inappropriate in use with a correlation of -0.541 and a withdrawal rate of inappropriate urinary catheters of 85.6%.

## Conclusions

The results show that low-cost educational interventions can reduce urinary infections and provide more security for patients in intensive care units.
